# Racialized Aging in the Context of Climate Extremes: Post-Flood Healthy Aging and Recovery Among Older Adults in Quilombola Communities of Southern Brazil

**DOI:** 10.3390/ijerph23030375

**Published:** 2026-03-17

**Authors:** Roberth Steven Gutiérrez-Murillo, Patricia Krieger Grossi, Gustavo Cezar Wagner Leandro, Márcio Lima Grossi

**Affiliations:** 1 School of Medicine, Pontifical Catholic University of Rio Grande do Sul, Avenida Ipiranga 6690, Prédio 81, Bairro Partenon, Porto Alegre 90619-900, RS, Brazil; pkgrossi@pucrs.br; 2 Department of Health Sciences, State University of Maringá, Avenida Colombo, 5790, Jardim Universitário, Maringá 87020-900, PR, Brazil; gustavocezarwl@gmail.com; 3 School of Life Sciences, Pontifical Catholic University of Rio Grande do Sul, Av. Ipiranga, 6681-Prédio 6, Partenon, Porto Alegre 90619-900, RS, Brazil; mlgrossi@pucrs.br

**Keywords:** healthy aging, Quilombola communities, natural hazards, environmental justice, disaster governance, quality of life, vulnerable populations, flooding

## Abstract

**Highlights:**

**Public health relevance—How does this work relate to a public health issue?**

- Extreme flooding directly threatens healthy aging by disrupting continuity of care, functional independence, social participation, and safe living environments for older adults in Quilombola communities.
- The study puts flooding into context as a critical social determinant of healthy aging, showing how environmental injustice and territorial exclusion accelerate functional decline and health deterioration in later life.

**Public health significance—Why is this work of significance to public health?**

- The research expands healthy aging knowledge by demonstrating how climate vulnerability and disaster governance failures undermine the ability of older adults to age with dignity, autonomy, and wellbeing in historically marginalized territories.
- By centering older Quilombola adults’ post-disaster quality of life, the study addresses a major evidence gap at the intersection of aging, climate change, and racial health inequalities.

**Public health implications—What are the key implications or messages for practitioners, policy makers and/or researchers in public health?**

- Healthy aging policies and programs must incorporate disaster risk reduction and climate adaptation strategies that protect functional capacity, ensure continuity of care, and support place-based aging for older adults in vulnerable communities.
- Public health practitioners and researchers should adopt equity- and rights-based approaches that recognize older adults as key stakeholders in disaster prevention and recovery, in addition to addressing structural determinants shaping unequal aging trajectories under climate change.

**Abstract:**

Background: Quilombola communities, Afro-descendant Brazilian rural settlements with collectivistic culture, have suffered historical invasions and non-legalization of their territories, exposure to environmental degradation/hazards, and educational and health care deprivation by the government. Global climate changes have increased sea levels and the occurrence of floods. This study presents original empirical findings from ongoing qualitative fieldwork in Quilombola communities in Southern Brazil that were severely affected by the 2024 floods, focusing on post-disaster quality of life, health impacts, and community coping strategies. These dimensions remain underexamined in public health and environmental justice research. Methods: Guided by interdisciplinary frameworks of environmental racism, intersectionality, and critical disaster studies, flooding is analyzed not as a natural hazard, but as a socially produced risk shaped by racialized territorial exclusion, historical marginalization, and chronic governance failures. Data were generated by household testimonies, community observations, and assessments of governmental disaster responses. Results: Fragmented disaster management, unequal access to infrastructure, and limited participatory governance mechanisms intensified vulnerability, constrained adaptive capacity, and exacerbated health inequities among Quilombola populations. Despite these constraints, communities demonstrated strong resilience grounded in traditional knowledge, local solidarity networks, and collective agency. Conclusions: The study underscores the urgent need for equity-centered environmental governance and inclusive disaster risk reduction strategies to address healthy aging inequities.

## 1. Introduction

Quilombola communities, Brazilian Afro-descendant rural populations with historical ties to enslaved resistance settlements [1], experience disproportionate exposure to environmental hazards due to intersecting processes of racialized territorial marginalization and structural neglect [2]. These territories are extensively threatened by extraction projects, overlapping private claims, and large infrastructure works, placing nearly all communities at risk of environmental degradation that undermines livelihoods and ecosystems essential to wellbeing [3]. More than 98% of Quilombola areas are currently under pressure from mining requests, land conflicts, and deforestation pressures that degrade natural systems and erode community resilience to climatic stressors [3].

In addition to these chronic pressures, global extreme weather events have increasingly impacted Quilombola populations. Between late April and May 2024, the Brazilian state of Rio Grande do Sul experienced a series of extreme weather events, characterized by heavy rainfall that resulted in unprecedented levels of flooding. This event overwhelmed the state’s existing flood defenses, affecting a significant portion of the region. A technical analysis presented by the Pan American Health Organization (PAHO) revealed it to be the most significant flood disaster that has ever occurred [4]. The event has been characterized by heavy precipitation, which has been linked to climatic anomalies such as El Niño, and exacerbated by infrastructural vulnerabilities. The floodplain of the Guaíba River Basin reached maximum water levels that have not been observed since 1941, with approximately 2.4 million individuals in 478 municipalities being directly impacted by flooding, displacement, and infrastructure collapse [5]. Estimates indicate that hundreds of thousands of homes were damaged or destroyed, more than 173 people died, and hundreds of thousands were displaced, with emergency shelters housing tens of thousands of flood survivors [6]. Specifically, for the Quilombolas, the intense rains and catastrophic flooding have isolated communities in Rio Grande do Sul [7], with reports indicating that multiple quilombos were cut off from transport and basic services for extended periods, disrupting access to health care and essential resources [8]. Such events exemplify the broader trend of climate-related hazards, including floods, droughts, and vector-borne disease risk, disproportionately affecting historically marginalized groups and exacerbating health inequities by damage to infrastructure, loss of housing, and interruption of medical care [9].

In addition to these exposures are longstanding socioeconomic and service access barriers documented across Quilombola territories, including limited sanitation [2], absent or distant health services [10], and precarious infrastructure [11] that magnify the health consequences of environmental shocks. These conditions produce cumulative vulnerabilities among older adults who already face higher burdens of chronic disease and functional limitations [12,13,14], illustrating how environmental hazards and institutional neglect intersect to shape differential health risks in aging Quilombola populations.

In this study, we advance our understanding of climate-related disasters and healthy aging by integrating insights from Critical Gerontology [15,16] and Environmental Justice [17] to examine the impacts of flooding in older adults from Quilombola communities in Southern Brazil. Critical gerontology highlights aging as a socially and politically produced process shaped by cumulative life-course inequalities, rather than biological decline, while environmental justice emphasizes the racialized and territorialized distribution of environmental risks and state protection. In complementing these perspectives, we conceptualize flooding not as an external shock acting upon a uniformly vulnerable older population, but as a socially produced hazard that interacts with historical marginalization, racialized land dispossession, and governance failures to shape unequal aging trajectories. This integrated framework enables us to state the following specific objectives:

1. Analyze the way that the 2024 flooding affected key dimensions of healthy aging among older adults in Quilombola communities.
2. Assess how the environmental injustice (i.e., conceptualized here as social due to its impact on the quality of life of vulnerable individuals), life-course inequalities, and disaster governance processes intersected to produce differential health impacts and recovery trajectories following the flood.
3. Evaluate what coping strategies, forms of collective organization, and traditional knowledge mobilized by Quilombola older adults contributed to wellbeing and resilience in the aftermath of the flood, and the way these insights informed more equitable public health and disaster risk reduction strategies that support healthy aging in climate-vulnerable territories.

Ultimately, we approach this work with a commitment to accountability, recognizing older Quilombola adults not only as research participants but as knowledge holders and rights-bearing subjects. Findings are intended to support community advocacy, to inform equitable public health and disaster governance, and to contribute to broader efforts to advance healthy aging and environmental justice under conditions of climate change.

## 2. Materials and Methods

### 2.1. Design and Theoretical Framework

We employed a qualitative-dominant mixed-methods design [18] grounded in critical gerontology and healthy aging perspectives. The study focused on post-disaster quality of life, health impacts, and coping strategies among older adults in Quilombola communities affected by the 2024 floods in the state of Rio Grande do Sul, Brazil.

Healthy aging is commonly defined as the process in which people maintain the physical, mental, and social capacities that allow them to live well in later life [19]. Although this concept has helped move aging research beyond narrow disease-based models, it often reflects assumptions rooted in urban, biomedical, and individual-centered contexts. From our empirical experience in Quilombola communities, growing older is closely tied to ancestral land, shared histories of resistance, and communal forms of care that extend beyond formal health systems [20]. Accordingly, we agree with Lowsky and Olshansky [21] when they posit that standard healthy aging frameworks and indicators do not always capture these dimensions and may inadvertently frame structural exclusion, such as limited access to services or environmental degradation, as individual decline. Admitting these limitations, we rely on a sensitized and context-aware approach to healthy aging, one that values local knowledge, collective wellbeing, and place-based forms of resilience. 

### 2.2. Ethical Clearance

Ethical and institutional approval was received from the Research Ethics Committee at the Pontifical Catholic University of Rio Grande do Sul, under the Certificate of Presentation for Ethical Consideration (CAAE #85437324.6.0000.5336, review #7319527), approved on 27 December 2024. 

We followed the Brazilian ethical guidelines for research with human subjects (CNS Resolution #510/2016) and protocols for research with traditional communities, as highlighted by Bosi and Guerreiro [22]. Informed consent was obtained orally or in writing, depending on participant preference. Interviews were conducted using trauma-informed approaches, with the option to pause or discontinue at any time. Participants requiring health or psychosocial support were referred to local services when available. Community leaders were informed of study objectives, and findings were shared with participating communities in 2026 upcoming meetings.

### 2.3. Setting and Population

According to the Brazilian Institute of Geography and Statistics (Instituto Brasileiro de Geografia e Estatística—IBGE) 2022 Census [23], there are 8441 Quilombola localities across the Brazilian territory, which are associated with 7666 Quilombola communities as reported by informants. “Localities” in this context are defined as places with a permanent cluster of self-declared Quilombola residents and are linked to communities for statistical purposes. The Southern Region (comprising the states of Rio Grande do Sul, Paraná, and Santa Catarina) accounted for 304 Quilombola localities in the 2022 Census, representing approximately 3.6% of identified localities nationwide [23]. In Rio Grande do Sul alone, 203 Quilombola localities were identified [24].

Fieldwork was conducted between March and September (2025) in Quilombola communities located in flood-affected areas of Porto Alegre, the state capital of Rio Grande do Sul [4]:

▪ Quilombo do Areal da Baronesa. This historically significant urban quilombo is located at Avenida Luís Guaranha in the Cidade Baixa/Menino Deus neighborhood in Porto Alegre. It has a long cultural visibility and has been recognized by Fundação Cultural Palmares and municipal authorities; its location is in low-lying urban zones exposed to recurrent flooding risk.
▪ Quilombo dos Machado. This quilombo in the Sarandi neighborhood in Porto Alegre has been documented as an active site of community support and mutual aid during flood events and broader social crises.
▪ Comunidade Família Lemos. Located in the Menino Deus/Av. Padre Cacique neighborhood in Porto Alegre, this community has ongoing territorial recognition processes and is part of the post-flood assistance mapping.
▪ Quilombo Fidelix. Situated in the Azenha neighborhood in Porto Alegre, it is another historically recognized community with documented socio-territorial claims that predates the floods.
▪ Quilombo dos Alpes and Quilombo da Família Silva. These communities in Porto Alegre, which are recognized or are in the process of territorial identification, together with Areal, Lemos, and Fidelix, represent the core recognized urban quilombos in the city.

Although Brazilian Senior legislation (Estatuto da Pessoa Idosa) defines older adults as individuals aged 60 years and over [25], this study included Quilombola adults aged 55 years and older. This threshold was adopted in recognition of their heightened vulnerability to disaster-related health impacts and their central role in sustaining community life, collective memory, and care networks.

### 2.4. Sampling and Recruitment

A purposive sampling strategy [26] was used to capture diversity in age, sex, functional status, displacement experience, and degree of flood exposure. Participants were identified through collaboration with Quilombola community leaders, local associations, and primary health care or social assistance networks. Inclusion criteria were: (a) self-identification as Quilombola; (b) age ≥ 55 years; and (c) direct experience of the 2024 flooding. Recruitment continued until thematic saturation was reached [26].

### 2.5. Data Collection

Data were generated by multiple complementary methods to capture both individual and collective dimensions of post-flood healthy aging. We conducted structured and semi-structured interviews using the validated Brazilian Portuguese World Health Organization Quality of Life-Brief Version (WHOQOL-BREF), which has shown a good performance concerning internal consistency, discriminant validity, criterion validity, concurrent validity, and test–retest reliability [27]. It is a self-report questionnaire designed by the WHO that measures an individual’s perception of their quality of life across four domains (i.e., physical, psychological, social, and environmental) [27]. The WHOQOL-BREF with 12 questions (Table A1), along with a Quilombola Flood Impact and Public Governance self-elaborated module, was used. Items were scored on a 5-point Likert scale, with higher scores indicating better perceived quality of life. This module was developed specifically for the purpose of this research to assess the flood exposure, displacement, disruption of health care and medications, access to governmental aid, participation in recovery processes, and perceptions of justice and future risk (Appendix A.1; Table A1). Both instruments were administered orally to accommodate varying literacy levels.

By in-depth narrative interviews, we explored older adults’ lived experiences of aging during and after the flood, focusing on functional capacity, autonomy, social participation, place attachment, loss, and coping. Open-ended prompts encouraged storytelling and reflection, allowing participants to define what “living well” and “aging with dignity” meant in their own terms:

1. What was your life like during and after the flood?
2. How did your health and daily life change after the flood?
3. What hurt the most to lose?
4. Who helped you getting through this difficult time?
5. What did the government do, and what did it fail to do?
6. What would it take to live better and safely?

Additionally, participatory mapping exercises were conducted individually to identify flooded areas, safe and unsafe spaces, damaged infrastructure, and access to health and social services. Field observations documented housing conditions, environmental degradation, community organization, and ongoing recovery efforts. Detailed field notes were maintained throughout the research process by the first author.

### 2.6. Data Analysis

Regarding the WHOQOL-BREF instrument, domain scores were analyzed descriptively, and lower scores were interpreted as indicating greater impairment or dissatisfaction within the respective domain [27]. Physical and psychological domains were examined as indicators of functional autonomy, emotional distress, and the capacity to age with dignity following environmental disruption. The social relationships domain was interpreted as reflecting collective care, social cohesion, and informal support networks, which are central to wellbeing in Quilombola communities. The environment domain was analyzed as a proxy for structural conditions, including housing security, perceived safety, access to health services, and disaster governance. This allowed quality of life outcomes to be interpreted by means of a life-course and environmental justice lens shaped by cumulative inequality and territorial marginalization. A plain characterization of WHOQOL-BREF domain-level variables can be found in Appendix A.2 (Table A2). 

Interviews were audio-recorded, transcribed *verbatim* from Brazilian Portuguese to American English, and analyzed using thematic analysis supported by NVivo (Release 1/v.2020) [28], a multilevel qualitative data analysis software. Interviews lasted between 39 and 51 min, with a mean of 44 min. An initial coding framework was developed deductively from the study’s theoretical lenses (i.e., healthy aging, environmental injustice, critical gerontology) and inductively refined by close reading of the data. Particular attention was paid to intersections of age, race, territory, and governance in shaping healthy aging outcomes.

Methodological rigor was ensured by triangulation of data sources, iterative team discussions, and reflexive memo-writing by the first and second authors. All excerpts were anonymized (Q_1_…Q_32_; “Q” meaning Quilombola) and presented as illustrative quotations to reflect recurring themes across participants, preserving confidentiality, while foregrounding lived experience. The analytical framework resulting from the interviews is fully introduced in Appendix A.3 (Table A3).

### 2.7. Positionality Statement

This research was conducted by an interdisciplinary team of public health and social science researchers with training in environmental justice, aging, and qualitative methods. We acknowledge that we did not share the lived experience of being older Quilombola residents affected by the 2024 floods; instead, we did occupy positions of relative social, institutional, and epistemic privilege. Also, we recognize that research in historically marginalized, and disaster-affected communities carries inherent power asymmetries and risks of extractive knowledge production.

To address these dynamics, our study was designed using community-engaged and reflexive approaches, prioritizing the voices, knowledge systems, and lived experiences of older Quilombola participants. Data collection emphasized oral, relational, and narrative methods consistent with local epistemologies and active aging practices. Community leaders and local actors were consulted during study planning and fieldwork to ensure cultural relevance, ethical conduct, and contextual accuracy. Throughout the research process, our team engaged in reflexive practices to critically examine how assumptions related to race, age, territory, and governance shaped research questions, interactions, and interpretation of findings. 

## 3. Results

### 3.1. Participant Profile

As presented in Table 1, the sample reflects an aging population marked by advanced age and pronounced social vulnerability, with more than one quarter of participants aged 75 years or older and over two thirds aged 65 and above. Women constituted a slight majority of the sample, consistent with broader demographic patterns of aging in Brazil. Educational attainment was notably low, with many participants reporting no formal schooling, underscoring the long-standing educational exclusion experienced by Quilombola populations and its implications across the life course.

Economic conditions were similarly constrained. For instance, most participants lived in households with monthly incomes of one to two minimum wages, and nearly three quarters depended primarily on retirement benefits or the BPC, proving the central role of social protection policies in sustaining livelihoods in later life for socially marginalized groups. Despite this reliance on income transfers, material vulnerability was acute: more than four fifths of households experienced severe housing damage because of the flooding, and all participants were displaced for extended periods, with three quarters remaining displaced for at least 20 days.

Access to health services following the disaster was widely compromised. A substantial majority of participants reported difficulties accessing the SUS in the post-disaster period, pointing to disruptions in continuity of care at a time of heightened health needs. Taken together, these findings illustrate how extreme flooding intersected with entrenched structural inequalities to intensify precarity, disrupt care networks, and further constrain the possibilities for healthy aging in Quilombola territories.

### 3.2. Quilombola Flood Impact and Public Governance

Older Quilombola participants described the 2024 flooding not only as a sudden environmental event, but as a profound rupture in their relationship with home, land, and community. Flood exposure and territorial loss were narrated as experiences of both material devastation and symbolic dispossession: “*My whole life is here. I was born here, raised my children here. When the flood came, it felt like it was pushing us out, like saying this place is no longer for us*” (Q02). A few participants emphasized the destruction of their homes, prolonged displacement, and the loss of subsistence spaces such as boonies, fields, and small animal holdings, which are central to daily autonomy and food security in later life: “*We don’t just live in the house. We live in the yard, in the river, in the path we walk every day. The flood took all of that at once*” (Q12). The flooding of cemeteries, rivers, and community churches was repeatedly described as especially painful, as these spaces anchor collective memory and intergenerational continuity. As one participant explained, “*It wasn’t just the house that filled with water. It was the place where my parents are buried, where we pray, where we know who we are. When that goes under, it feels like we’re being erased*” (Q23). 

Health and care disruption emerged as a critical pathway by which the disaster affected quality of life. Participants reported losing medications, prescriptions, and assistive devices during the floods, alongside major difficulties accessing health services during displacement: “*Just look at me, you see, I use a cane. In the shelter, I couldn’t walk properly and felt afraid of falling all the time*” (Q22). Chronic conditions such as hypertension, diabetes, and mobility limitations were frequently described as worsening in the weeks that followed. One older woman noted, “*I had to leave with only the clothes on my body. My pills stayed behind. After that, my pressure went out of control, and there was no doctor, no health unit to help us*” (Q28). These disruptions were experienced as particularly destabilizing for older adults who depended on regular care and daily routine to maintain health and functional autonomy: “*We were moved far away. To see a doctor, we needed transportation, documents, and patience. Most of the time, we had none of that because of the flooding*” (Q15).

Changes in social support and care networks revealed both vulnerability and resilience. While many participants became more dependent on family members or neighbors for daily activities, they also emphasized the strength of community solidarity in the absence of effective institutional support: “*Following the flood, I needed help to bathe, to cook, to walk, because I ended up breaking my right leg. I still do, but not that much, you know? Depending on others at my age is very hard on the heart*” (Q29). Mutual aid, shared food, and collective caregiving were repeatedly cited as essential to survival and emotional wellbeing: “*I must be honest, man, if it wasn’t for my neighbors, I don’t know how I would have managed. We shared food, space, and care like family*” (Q31). At the same time, some participants described moments of loneliness, bereavement, and abandonment during displacement. As one man reflected, “*If it wasn’t for the people here, we would have been lost. But there were nights when I felt very lonely, not knowing when we could go back or if anyone remembered us. I lost my daughter. They gave us no information about our family whereabouts during the first week*” (Q04).

Perceptions of state response and governance were largely marked by frustration and a sense of invisibility: “*Well, being Quilombola already makes things harder. After the flood, it became even more clear who is seen and who is not*” (Q30). Participants described delays in emergency aid, bureaucratic obstacles to accessing benefits, and a lack of culturally appropriate engagement with Quilombola communities: “*We took well care of each other. The government didn’t arrive, but the community did. This is when you see who really matters”* (Q14). Several emphasized that decisions about recovery were made without their participation: “*They came, took pictures, wrote things down, and left. Nobody asked us what we needed or how we live here*” (Q21); “*This flood didn’t happen by chance. The river was changed, the land was destroyed. We’ve been saying this for years*” (Q13).

Finally, perceived justice and future risk reflected a critical awareness of structural causes and ongoing vulnerability. Many participants believed that the impacts of the flooding could have been reduced by better environmental management, infrastructure, and respect for Quilombola territories. In particular, we noticed widespread concern about future floods and uncertainty about whether it would be possible to age safely in place. As one participant summarized, “*This water didn’t come alone. It came because they don’t care for the land or for us. They never have. And let me tell you, if nothing changes, it will come again, and we will be older*” (Q32). Across interviews, participants consistently framed flooding as both an environmental event and a social injustice, linking material losses, health deterioration, and emotional distress to long-standing patterns of territorial neglect and exclusion.

### 3.3. Quality of Life Post-Flooding

WHOQOL-BREF findings indicated substantially compromised quality of life among older Quilombola adults following the 2024 flooding in Rio Grande do Sul (Table 2). Overall quality of life and satisfaction with health were low, with mean scores of 2.3 and 2.1, respectively, reflecting widespread dissatisfaction in the post-disaster context. The physical health domain showed poor overall performance (mean = 2.4), characterized by frequent pain interfering with daily activities, low energy levels, and reduced functional autonomy and work capacity. Psychological wellbeing was similarly affected (mean = 2.3), with participants reporting diminished enjoyment of life and frequent negative emotions, including anxiety, sadness, and distress, consistent with prolonged displacement and uncertainty following the floods.

In contrast, the social relationships domain showed comparatively higher scores (mean = 3.5), indicating moderate satisfaction with personal relationships and strong perceived support from family and community networks. This pattern suggests a protective role of informal care and collective solidarity in mitigating some of the adverse impacts of the disaster. The environment domain was the most severely compromised (mean = 2.0), with low perceived safety and pronounced dissatisfaction with access to health services, reflecting extensive housing damage, prolonged displacement, and disruptions to the SUS (Table 2).

### 3.4. Lived Experiences of Aging During and After the Flood

Participants consistently described the flood as a moment of abrupt rupture that disrupted daily routines, sense of safety, and continuity of life in later age. During the flood, life was characterized by fear, confusion, and forced displacement; in its aftermath, many spoke of prolonged instability, uncertainty, and difficulty re-establishing everyday rhythms. Aging during the disaster was experienced as particularly disorienting, as familiar spaces that supported autonomy and memory were suddenly inaccessible: “*How can I explain it? One day I was living my normal life, the next day I was running from the water. After that, nothing went back to the way it was*” (Q25); “*After the flood, life felt suspended. We were alive, but not living yet”* (Q01).

Health impacts were described as both physical and emotional, often intertwined with loss of routine and independence. Participants reported worsening of chronic conditions, fatigue, pain, sleep disturbances, and emotional distress. Daily activities that once sustained autonomy, such as cooking, caring for animals, or tending gardens, became difficult or impossible, increasing dependence on others: “*I think that my body got weaker. I feel more pain now, more tired. Without my routine, my health went down. People around me tell so, too*” (Q16); “*I used to take care of myself. But with the flood, I saw myself needing help for everything. That was frustrating, really*” (Q23).

While material losses were significant, participants emphasized that the most painful losses were tied to identity, memory, and belonging. Homes, fields, animals, and community spaces were pictured as extensions of the self and of collective history: “*Furniture can be replaced, for sure. What really hurts is losing the place where my life happened*” (Q08). The loss of these spaces was experienced as a form of emotional and cultural dispossession: “*I lost my yard, my plants, my memories. That’s what hurts the most*” (Q29).

Support came primarily from family members, neighbors, and the Quilombola community itself. Mutual aid, shared caregiving, and collective problem-solving were repeatedly highlighted as central to survival and emotional endurance. Participants acknowledged limited emergency actions, but overwhelmingly emphasized institutional absence, delays, and lack of culturally appropriate engagement. Many voiced that government responses were insufficient and disconnected from Quilombola realities: “*They never asked us what we needed. We did not expect differently now with these floods, they decided from far away*” (Q16).

Living better and aging healthily, with dignity, were framed not only as individual needs but as collective and structural conditions. Participants emphasized the importance of territorial protection, flood prevention, access to health care, respect for Quilombola identity, and being heard in decisions that affect their lives. Healthy aging was closely tied to the possibility of remaining in their territory with adequate support: “*It isn’t that complicated. To live better, we need protection for our land and respect for who we are. We don’t ask for more than what we deserve*” (Q02); “*They need to understand it already [public authorities]. Aging with dignity means staying here, safely, without fear of losing everything again. How many other floods are needed so that we can get the support we need?*” (Q32).

Our field observations conducted in the aftermath of the 2024 flooding revealed a landscape of profound environmental and infrastructural destruction that shaped older Quilombola adults’ everyday lives and recovery. Streets within the communities remained covered with mud, debris, and waste, often fractured or impassable, while damaged drainage systems and persistent standing water transformed familiar paths into sources of risk. Many houses showed visible structural damage, including collapsed walls, compromised roofs, and high-water marks, with interiors stripped of furniture, food, and personal belongings. Notably, for older residents, these material losses translated into prolonged displacement, as homes remained unsafe or uninhabitable weeks after the waters receded.

Moreover, temporary living arrangements frequently lacked privacy, accessibility, and adequate conditions for rest, compounding physical discomfort and emotional strain. Mobility was severely constrained, with older adults observed navigating unstable ground or relying on assistance to move within the community, limiting access to health services, markets, and social spaces. The slow pace of cleanup and infrastructure repair, alongside the limited presence of public authorities, made visible long-standing patterns of neglect in Quilombola territories.

## 4. Discussion

### 4.1. General Findings of the Study

In this study, we bring novel empirical insights into how extreme flooding intersects with aging, race, territory, and governance to shape health and quality of life among older Quilombola adults in Southern Brazil. We demonstrated, by combining standardized quality-of-life measures with in-depth narrative interviews and field observations, that the impacts of the 2024 flooding extended far beyond immediate physical damage, producing cascading effects on functional capacity, autonomy, continuity of care, and the ability to age with dignity in place. Our findings challenge dominant framings of disasters as short-term natural events; instead, we highlight flooding as a socially produced risk that amplifies long-standing structural inequalities.

### 4.2. Public Responses to the Flood

Consistent with critical gerontology horizons [29,30], especially those of environmental gerontology framed within a life-course perspective [31,32], our analyses showed that older Quilombola adults entered the disaster with accumulated vulnerabilities, which were determined by longstanding exposure to racialized exclusion, low educational attainment, economic precarity, and infrastructural neglect. Admittedly, the 2024 flooding acted as a tipping point that accelerated health decline, particularly by disrupting routines and resources that support functional autonomy in later life. Low scores in the physical and psychological domains of the WHOQOL-BREF reflected not only injury or illness, but also the embodied consequences of displacement, loss of territory, and uncertainty. These results align with national [33,34,35] and international evidence [36,37] indicating that flooding disproportionately affects older adults by undermining the environmental and social conditions necessary for healthy aging, especially amid Indigenous and Quilombolas communities.

In the immediate aftermath of the historic floods, the Government of Rio Grande do Sul declared, under Governor Eduardo Leite, a 180-day state of emergency and mobilized a broad response effort to address urgent needs; in addition, it began a long-term recovery, recognizing the unprecedented scale of the disaster and enabling expedited use of resources for response and relief efforts. In May 2024, the state launched the Plano Rio Grande, a comprehensive program to support emergency relief, infrastructure reconstruction, and resilience building [38]. This plan coordinated multiple lines of action, from immediate humanitarian support to medium- and long-term investments in public services and disaster preparedness. Moreover, the State Public Archive began a project to document the “Memories of the 2024 Floods”, capturing public administration actions and experiences during and after the disaster to inform future preparedness and historical knowledge [39].

Although these responses represented massive mobilization and investment, mainly in physical infrastructure and disaster governance, community narratives and field data suggest persistent gaps in inclusive engagement, culturally appropriate support, and timely access to care and services. This juxtaposition highlights the need for future policy responses that not only rebuild physical infrastructure, but also strengthen equitable access to health services, social protection, and participatory governance for Quilombola communities. As a multidimensional public concern, it requires an intersectoral approach, as flooding disproportionately impacts populations living in poverty, in low-development contexts, and in territories shaped by persistent income inequality [40].

The environmental damage documented by field observations (i.e., collapsed housing, destroyed streets, persistent contamination, and slow infrastructure repair) further illustrates how environmental injustice becomes materially inscribed in Quilombola territories [41]. Aging in place, widely promoted as a healthy aging and public health goal [42], was rendered unattainable for many Quilombolas due to unsafe housing, impaired mobility, and disrupted access to services. The loss of subsistence spaces and culturally significant sites further eroded older adults’ sense of belonging and identity, reinforcing evidence that place attachment is a critical but often overlooked determinant of wellbeing in later life [43]. As we see it, such findings suggest that healthy aging frameworks fail to account for territorial and environmental dimensions and risk excluding traditional communities, whose health is inseparable from land and collective memory.

Disruptions in access to medications, assistive devices, and rehabilitative therapies following the floods exposed significant gaps in disaster preparedness within the SUS for older populations. Although not explicitly reported by participants, infectious disease risks are well documented in the aftermath of flooding events and represent a significant public health concern [44]. They can facilitate the transmission of waterborne, vector-borne, and respiratory infections, which may escalate into outbreaks if not promptly and adequately addressed. In that vein, narratives of delayed or inaccessible care highlight the fragility of continuity-of-care models during climate-related emergencies, mirroring outcomes observed after the 2015 dam failure in the Quilombola community of Degredo, Minas Gerais [41]. From a governance standpoint, the limited inclusion of Quilombola communities in recovery planning and decision-making processes reflects broader patterns of institutional neglect and environmental racism. Therefore, we understand these as persistent failures that not only constrain recovery, but also undermine trust in public institutions, with implications for future risk communication and disaster response effectiveness.

### 4.3. Community Response to the Flood

Despite these challenges, our findings also highlight the central role of community solidarity, informal care networks, and traditional knowledge in sustaining wellbeing during and after the disaster, an expected scenario within Quilombolas communities [45]. Social relationship scores in the WHOQOL-BREF were comparatively higher, reflecting the protective effects of mutual aid and collective caregiving. However, reliance on informal networks should not be interpreted as a substitute for State liability. Rather than that, public health interventions should build on these strengths by supporting community-led preparedness and recovery efforts, ensuring that resilience does not become a justification for continued institutional neglect.

The lived experiences of interviewees also suggest that healthy aging is inseparable from the health of broader social and ecological systems. Hence, aging well in Quilombola territories cannot be understood solely through individual health indicators, but rather through a One Health perspective [46] that recognizes the interdependence of human, environmental, and animal wellbeing. The floods produced not only material and financial losses, but also profound ecological disruption, affecting soils, water sources, animals, and subsistence practices. Within Quilombola cosmovision, environmental degradation and animal suffering are understood as direct threats to collective health and dignity [17], dimensions that are frequently overlooked by public authorities in disaster response and recovery efforts. Even in urbanized areas of Porto Alegre, the communities we visited articulated a deep and enduring relationship with the land, grounded in practices of care, reciprocity, and coexistence with nature. These relational ties help explain how aging is experienced and give meaning to wellbeing in later life, calling for public policies that protect ecosystems and territories as a fundamental component of promoting healthy aging.

Noteworthily, by integrating critical gerontology with environmental justice, this study advances a framework for understanding disasters as life-course events that reconfigure aging trajectories in racially and territorially marginalized contexts. Flooding emerges not merely as an environmental exposure, but as a governance failure that disproportionately harms older adults whose lives are deeply rooted in place. This theoretical integration highlights the need for equity-centered disaster risk reduction strategies that recognize older adults as rights-bearing subjects, with valuable knowledge and leadership capacities, rather than situating them as passive victims. For instance, aligning disaster risk reduction strategies with the Sendai Framework [47] and Brazil’s National Policy for the Health of Older Persons [48] can help to ensure that climate adaptation efforts address both immediate hazards and the structural conditions that shape vulnerability across the course of life.

### 4.4. Implications for Public Health and Future Environmental Disaster Policies

Our findings have important implications for public health and disaster governance in Brazil. Promoting healthy aging across Quilombola communities requires age-sensitive disaster preparedness within SUS, including mobile health teams, medication continuity protocols, and accessible shelter designs. Territorial protection, infrastructure investment, and meaningful participation of Quilombola communities in environmental governance are essential to reduce future risks [31]. To prevent avoidable suffering and support aging with dignity in climate-vulnerable territories, coordinated action is urgently required, especially when considering environmental racism and governance gaps [49,50].

One important area for improvement is getting institutions to recognize and strengthen community-led networks of mutual support and care as official parts of disaster preparedness and healthy aging strategies. The openness, reciprocity, and sense of brotherhood shown by Quilombola communities during the floods worked as powerful protective factors. They made it easier to provide informal care, share resources, give emotional support, and respond quickly when formal systems were delayed or absent. Public health systems can build on this strength by partnering with community leaders and elders, supporting locally organized care brigades, integrating community knowledge into emergency planning, and providing technical, logistical, and financial support without undermining community autonomy. These social networks are important, because they help people to cope with disasters and promote dignity and wellbeing in later life. They also encourage healthy aging by building community resilience, which, as participants stated, is better than relying solely on institutional support.

### 4.5. Limitations and Suggestions for Future Studies

This study is not without limitations, and they should be understood not as weaknesses, but as reflections of the realities in which Quilombola communities live and age. While the number of participants was limited to 32 individuals and the research focused on specific territories, the experiences documented here echo long-standing structural conditions faced by Quilombola and other traditional communities across Brazil. In contexts shaped by historical exclusion, where official data are often scarce or incomplete, listening closely to communities and documenting their lived experiences becomes essential for making inequalities visible and informing fairer public policies. Because this study captured a specific moment shortly after the disaster, it reflects the period when loss, uncertainty, and institutional gaps were most acutely felt. Although it does not follow participants over time, the evidence clearly reveals how disruptions to housing, health care, and social protection unfold in the immediate aftermath of flooding, precisely the moment when older adults are most vulnerable and when timely public action can prevent avoidable suffering.

The use of self-reported data was a deliberate and meaningful choice. Allowing older adults to describe their own health, safety, and sense of dignity by centering their voices in a context where decisions are often made without their participation. These narratives offer indispensable insight into where systems failed and where protections were insufficient, helping us to identify gaps that may otherwise remain invisible in administrative records. Ultimately, the absence of a comparison group reflects the ethical and practical challenges of conducting disaster research in marginalized settings. When consistent patterns of harm are observed among populations already exposed to cumulative disadvantages, waiting for perfect data before acting risks reproducing injustice. From a public health and human rights perspective, such evidence strengthens the case for precautionary, protective, and preventive policies that prioritize those most at risk. 

Future studies should be performed using larger samples and should be multicenter to assess the consequences of future environmental tragedies in Brazil as a whole, not only in one state, due to sharp regional differences. 

## 5. Conclusions

The 2024 flooding in Rio Grande do Sul revealed how environmental injustice and governance failures can profoundly disrupt healthy aging among Quilombola communities. Addressing these challenges requires reimagining public health responses to climate-related disasters through lenses that center equity, territory, and the lived experiences of older adults. Only the integration of critical gerontology and environmental justice can develop effective strategies of disaster risk reduction, which also supports healthy aging in historically marginalized and climate-vulnerable territories.

## Figures and Tables

**Table 1:** Profile of older Quilombola adults participating in the study, affected by the 2024 flooding in Rio Grande do Sul, Brazil (N = 32).

Variable	Category	*n* (%)
Age group (years)	55–65	13 (40.6)
	66–74	10 (31.3)
	≥75	9 (28.1)
Sex	Female	17 (53.1)
	Male	15 (46.9)
Educational attainment	No formal schooling	25 (78.1)
	Incomplete primary	7 (21.9)
Monthly household income	≤1 minimum wage	8 (25.0)
	1–2 minimum wages	21 (65.6)
	3 minimum wages	3 (9.4)
Main source of income	Retirement/BPC	23 (71.9)
	Family support	7 (21.9)
	Informal work	2 (6.2)
Housing damage due to flooding	Severe	27 (84.4)
	Moderate	5 (15.6)
Displacement after flooding (days)	20–39	24 (75.0)
	≥40	8 (25.0)
Access to SUS post-disaster	Difficult	26 (81.2)
	Moderate	6 (18.8)

BPC = Benefício de Prestação Continuada (Continuous Payment Benefit). Minimum wage refers to the Brazilian national minimum wage in 2024. SUS = Sistema Único de Saúde (Brazilian Unified Health System).

**Table 2:** Descriptive responses of older Quilombola adults affected by the 2024 flooding (N = 32), according to the WHOQOL-BREF with 12 questions (post-flooding).

Domain	Question	Mean * (SD)	Interpretation
Overall QoL **	How would you rate your quality of life?	2.3 (0.8)	Poor–neither good nor poor
Overall Health	How satisfied are you with your health?	2.1 (0.7)	Dissatisfied
Physical health	To what extent do you feel pain prevents you from doing what you need to do?	3.9 (0.9)	Pain frequently limiting
	Do you have enough energy for everyday life?	2.2 (0.8)	Low energy
	How satisfied are you with your ability to perform daily activities?	2.4 (0.9)	Limited functional autonomy
	How satisfied are you with your capacity for work?	2.0 (0.8)	Markedly reduced
Psychological	How much do you enjoy life?	2.3 (0.7)	Reduced enjoyment
	How often do you have negative feelings (anxiety, sadness, despair)?	3.8 (0.8)	Frequent distress
Social relationships	How satisfied are you with your personal relationships?	3.4 (0.9)	Moderately satisfied
	How satisfied are you with the support you get from friends/family?	3.6 (0.8)	Strong informal support
Environment	How safe do you feel in your daily life?	2.1 (0.7)	Low perceived safety
	How satisfied are you with access to health services?	1.9 (0.6)	Very dissatisfied

* Values were calculated from a 1–5 Likert scale. ** QoL: Quality of life.

## Data Availability

Data presented in this study are available on reasonable request from the corresponding author. The data are not publicly available due to the sensitive and potentially identifiable nature of the information.

## Abbreviations

The following abbreviations are used in this manuscript:

SUS	Sistema Único de Saúde (Brazilian Unified Health System)
PAHO	Pan American Health Organization
IBGE	Instituto Brasileiro de Geografia e Estatística (Brazilian Institute of Geography and Statistics)
BPC	Benefício de Prestação Continuada (Continuous Payment Benefit)
WHO	World Health Organization

## Appendix A

### Appendix A.1

**Table A1:** Quilombola Flood Impact and Public Governance questionnaire, Rio Grande do Sul, 2025.

Domains	Questions
Flood Exposure and Territorial Loss	Was your home flooded during this disaster? (Partially/Completely/No) Did you have to leave your home? For how long? Was there loss of farmland, livestock, home gardens, or shared community-use areas? Was any important place in the community affected (cemetery, river, church, community association)?
Health and Care Disruption	Did you lose medications, prescriptions, or assistive devices (cane, wheelchair, glasses)? Did you have difficulty accessing a health clinic, hospital, or health care team after the flood? Did any health problem worsen after the flood? Which one(s)?
Social Support and Care Networks	Did you receive help from family members, neighbors, or the community? Did you become more dependent on someone for daily care after the flood? Did you feel lonely or unsupported at any point?
State Response and Governance	Did you receive any assistance from the government? (What type? When?) Did you have difficulty accessing benefits (documents, transportation, information)? Did you feel that the community was respected and listened to by authorities? Were you invited or able to participate in meetings or decisions about recovery?
Perceived Justice and Future Risk	Do you think the flood could have been prevented or its impacts reduced? Why? Do you think your community is more protected today than it was before the flood?

### Appendix A.2

Domain-level patterns below highlight how post-disaster quality of life among older Quilombola adults is shaped not only by individual health status, but also by social relations, territorial conditions, and institutional responses following extreme flooding of 2024.

**Table A2:** Characterization of WHOQOL-BREF Domain-Level Variables, Rio Grande do Sul-Brazil, 2025.

Domains	Contextualization
Physical Health	It captures self-reported aspects of functional capacity and bodily wellbeing, including pain and discomfort, energy and fatigue, mobility, ability to perform activities of daily living, and perceived capacity for work. In the post-flooding context, this domain reflects the extent to which disaster-related displacement, loss of assistive devices, interruption of medical treatment, and worsening of chronic conditions constrained participants’ physical autonomy and everyday functioning.
Psychological Health	It encompasses emotional wellbeing, life satisfaction, positive and negative affect, and perceived enjoyment of life. In this study, scores in this domain were interpreted as indicators of psychological distress and emotional burden associated with flooding, prolonged displacement, uncertainty, and loss of home and territory, as well as coping with aging under conditions of heightened environmental and social insecurity.
Social Relationships	It assesses satisfaction with personal relationships, availability of social support, and perceived quality of interpersonal interactions. For older Quilombola adults, this domain was used to characterize the strength and protective role of family ties, community solidarity, and informal care networks mobilized in response to the disaster, particularly in contexts where formal institutional support was limited or delayed.
Environment	It reflects perceptions of safety, housing conditions, access to health and social services, transportation, and broader living conditions. In the aftermath of flooding, this domain was interpreted as a proxy for structural and governance-related determinants of quality of life, capturing the material impacts of housing damage, barriers to accessing the SUS, perceived exposure to future environmental risks, and broader infrastructural and institutional shortcomings affecting recovery.

### Appendix A.3

Items in sections D and E explicitly operationalize dimensions of environmental injustice and disaster governance, including recognition, participation, distributive equity, and accountability. When analyzed alongside WHOQOL-BREF domains (Table A2), this module enables an integrated assessment of post-flooding quality of life, healthy aging, and structural vulnerability among older Quilombola adults.

**Table A3:** Analytical framework for the Quilombola Flood Impact and Public Governance module, Rio Grande do Sul, 2025.

Analytic Category	Core Meaning	Key Themes Captured
A. Flood Exposure and Territorial Loss	Direct experience of flooding as both a material and symbolic loss, affecting housing, livelihoods, and culturally meaningful spaces	→ Housing destruction and displacement → Loss of subsistence resources (roça, criação, quintal) → Damage to collective and sacred sites (cemetery, church, rivers) → Disruption of place-based identity and ability to age in place
B. Health and Care Disruption	Interruption of health maintenance and care continuity following the disaster	→ Loss of medications, prescriptions, and assistive devices → Barriers to accessing health services post-flooding → Worsening of chronic conditions → Increased health risks during displacement
C. Social Support and Care Networks	Changes in informal care, solidarity, and emotional wellbeing after flooding	→ Activation of family and community support networks → Increased dependency for daily care → Experiences of loneliness, abandonment, or emotional distress → Collective resilience and mutual aid
D. State Response and Governance	Perceptions and experiences of governmental action, recognition, and participation	→ Access to emergency aid and social benefits → Bureaucratic and informational barriers → Respect for Quilombola identity and territorial rights → Inclusion or exclusion from recovery-related decision-making
E. Perceived Justice and Future Risk	Interpretations of responsibility, preventability, and future vulnerability	→ Attribution of flooding to structural or environmental causes → Perceived preventability of disaster impacts → Trust in institutions and future protection → Anticipated climate risk and uncertainty about aging safely in the territory
